# Psychological Network of Stress, Coping and Social Support in an Aboriginal Population

**DOI:** 10.3390/ijerph192215104

**Published:** 2022-11-16

**Authors:** Pedro Henrique Ribeiro Santiago, Gustavo Hermes Soares, Lisa Gaye Smithers, Rachel Roberts, Lisa Jamieson

**Affiliations:** 1 Australian Research Centre for Population Oral Health (ARCPOH), Adelaide Dental School, The University of Adelaide, Adelaide 5000, Australia; 2 School of Public Health, The University of Adelaide, Adelaide 5005, Australia; 3 School of Health and Society, University of Wollongong, Wollongong 2500, Australia; 4 School of Psychology, The University of Adelaide, Adelaide 5000, Australia

**Keywords:** network psychometrics, stress, coping, social support, Aboriginal Australians

## Abstract

Over the past decades, increasing research interest has been directed towards the psychosocial factors that impact Aboriginal health, including stress, coping and social support. However, there has been no study that examined whether the behaviours, cognitions and emotions related to stress, coping and social support constitute a psychological network in an Aboriginal population and that examined its properties. To address this gap, the current study employed a new methodology, network psychometrics, to evaluate stress, coping and social support in an Aboriginal Australian population. This study conducted a secondary analysis of the South Australian Aboriginal Birth Cohort (SAABC) study, a randomised controlled trial in South Australia, which included 367 pregnant Aboriginal women at study baseline. The Gaussian Graphical Model was estimated with least absolute shrinkage and selection operator (LASSO). Node centrality was evaluated with eigencentrality, strength and bridge centrality. Network communities were investigated with the walktrap algorithm. The findings indicated that stress, coping and social support constituted a connected psychological network in an Aboriginal population. Furthermore, at the centre of the network were the troubles experienced by the Aboriginal pregnant women, bridging their perceptions of stress and coping and constituting a potential target for future interventions.

## 1. Introduction

Over the past decades, increasing research interest has been directed towards the psychosocial factors behind health inequalities experienced by Aboriginal Australians. As a consequence of the colonisation process that started in the late 1700s, Aboriginal Australians became a marginalised group within Australia [1]. The past and contemporary practices of land dispossession, colonial violence, child removal, mass incarceration, and institutional racism contribute to generating chronic stress, impacting social and emotional well-being, and perpetuating inequalities that disproportionately affect Aboriginal peoples in Australia [2,3]. As such, the stress and trauma experienced by Aboriginal Australians due to colonisation are not restricted to the first Aboriginal generations but persist throughout subsequent generations in a process named “intergenerational trauma” [4,5]. Intergenerational trauma occurs due to the collective experience of colonial injuries experienced by Aboriginal populations, whose identity, culture, ways of life and interactions were radically altered, and its cumulative effects on individuals from contemporary generations [6,7] (for an in-depth discussion on the transmission mechanisms of intergenerational trauma, please refer to Kirmayer, et al. [7]). Research has shown that the disruption of the social fabric of Aboriginal societies has brought contemporary Aboriginal peoples long-term psychological sequelae, such as a higher lifetime risk of depression or suicidality [8], physiological distress [9], and death from suicide compared to their non-Aboriginal counterparts [8,10]. The sustained and cumulative effects of stressful life events negatively impacts the social and emotional well-being of Aboriginal individuals [11] and add a significant burden to Aboriginal communities [3]. Among the psychosocial factors reported to influence Aboriginal health, the main factors are stress [12], coping [13], and social support [14]. It is important to note that Aboriginal communities have also demonstrated tremendous resilience and the ability to thrive despite the largely unfavourable conditions [15]. Community resources such as social support are likely to buffer the harmful repercussions of traumatic and stressful experiences on the social and emotional well-being of Aboriginal populations [14].

Given that the literature indicates that these factors (stress, coping, social support) impact Aboriginal health, it is fundamental to understand the dynamics of the complex psychological system that includes stress, coping, and social support, including understanding how it is possible to intervene in this system (e.g., whether increasing social support would decrease stress). To answer this research question, we will conduct a network analysis of stress, coping and social support in an Aboriginal population. In the next sections, we will describe the theoretical framework that underpins the conceptualisation of psychological networks (Section 1.1), which are complex psychological systems including behaviours, cognitions and emotions. We will then describe the analytical strategies employed to investigate psychological networks from empirical data (Section 1.2). Finally, we will discuss in detail the aims and research questions of the current study (Section 1.3).

### 1.1. Theoretical Framework of Psychological Networks

In psychology, the conceptual framework most traditionally used to understand and explain psychological processes is the latent variable theory [16]. The latent variable theory postulates that there is an unobservable psychological process that is the common cause of observable psychological processes such as behaviours, cognitions, emotions, attitudes, and symptoms of a mental disorder, among others (traditionally measured by the questionnaire items) [17]. For example, psychoanalytical traditions have attributed the stress response to unconscious neurotic processes [18], while neuroscience has investigated neuroanatomical substrates involved in the physiological changes associated with stress [19]. As such, according to the latent variable theory, a common cause or a relatively constrained set of causes (such as unconscious psychological processes, personality traits or dysfunctional brain circuits) are responsible for the observable behaviours, cognitions and emotions—and the behaviours, cognitions and emotions are ontologically distinct from the causes themselves [20]. For more than a century, this theoretical framework has been ubiquitous in psychological research, starting with the research work of Spearman [21] in 1904 postulating that the (unobservable) general factor (g-factor) of intelligence was the common cause of the different types of intelligence (e.g., musical, mathematical).

Given this theoretical framework, statistical models named latent variable models were then developed to model and explain psychological processes, such as factor analytical models (e.g., Exploratory Factor Analysis (EFA)), Structural Equation Models (SEMs) or item response theory models [16]. One main assumption shared across all these statistical models is that there are one or more “latent variables” that are the common cause of the behaviours, cognitions and emotions, leading the item responses to be marginally correlated in the data. The marginal correlations observed between item responses in the data are thus considered “spurious” (i.e., “confounded”), since the items should only correlate due to a shared common cause, the latent variable. The statistical implication of this assumption is that the item responses should be conditionally independent (should not be correlated anymore) once the influence of the latent variable is accounted for. Hence, in the theoretical framework of latent variable theory (and behind the use of latent variable models), behaviours, cognitions and emotions (measured by questionnaire items) are merely seen as indicators of the latent trait, which is considered of real scientific interest [22]. In fact, this theoretical understanding underpins the use of the term “symptoms” in psychology to refer to behaviours, cognitions and emotions associated with a mental disorder (e.g., “depressive symptoms” such as frequent crying, suicidal thoughts and feeling guilty). The term “symptoms” was borrowed from the 19th century medical sciences which achieved enormous scientific success by discovering bacteria to be the common cause of symptoms associated with a medical condition (e.g., Mycobacterium tuberculosis was the cause of tuberculosis and its symptoms, such as coughing up blood or mucus, chest pain, among others) [20,23]. Analogously, the use of the term “symptoms” in psychology implies that behaviours, cognitions and emotions are only indicators of an (unobservable) mental disorder that is the common cause of them, which exists independently of them [20] and which should be intervened upon (e.g., depression is a brain disorder that occurs due to chemical imbalances and the main intervention should be medications to restore normal serotonin levels) [24]. Due to this reason, psychologists who adhere to the network theory (discussed below) have debated against the use of the term “symptom” [20], the reason why we do not use the term in this manuscript to refer to behaviours, cognitions and emotions.

In research practice, this conceptualisation of behaviours, cognitions and emotions (measured by individual items) as mere indicators of the latent variable (of real scientific interest) led researchers to use total scores, subscale scores or factor scores (instead of item scores) to investigate and explain psychological processes [22]. Thus, psychological research has traditionally modelled the associations between latent variables (using total scores, subscale scores and factor scores) instead of the associations between behaviours, cognitions and emotions (item scores).

Research within the framework of latent variable theory (and using latent variable models) has been conducted to investigate stress, coping and social support among Aboriginal Australians. Previous studies had investigated stress, coping and social support separately (e.g., Waterworth, et al. [14] focused on social support) or examined the association between total scores of these constructs. For instance, Brown, et al. [12] showed that the total score of a social support measure was not a strong predictor of the total score of a depression measure. Another study demonstrated that total scores of psychological distress (measured with the Kessler psychological distress scale) were strongly associated with total scores of depression or anxiety [25].

Over the last decade, in contrast to the century-old latent variable theory, a new scientific paradigm has emerged to understand and explain psychological processes, named the network theory of psychological processes (or, briefly, network psychometrics). This framework started with the research work of Van Der Maas, et al. [26] in 2006 postulating that, instead of a general factor (g-factor), a network of mutually reinforcing cognitive processes could explain the positive correlations observed in the data between the different types of intelligence (e.g., musical, mathematical). Hence, within this new theoretical framework, behaviours, cognitions and emotions establish mutually reinforcing causal relations among themselves instead of being commonly caused by an unobservable latent trait [17]. The network psychometrics framework is theoretically consistent with areas of clinical psychology such as cognitive–behavioural therapy, in which psychological processes such as depression were already understood as mutually reinforcing causal relations between behaviours, cognitions, and emotions (e.g., difficult sleeping causes fatigue, fatigue causes sad mood, sad mood causes difficulty sleeping, and so forth) [20]. According to this new theoretical understanding, terms such as “depression” or “generalised anxiety” are merely verbal labels given to these complex psychological systems of mutually reinforcing causal relations established between behaviours, cognitions and emotions. That is, “depression” or “generalised anxiety” are not comprehended as unobservable (unconscious or biological) processes that are the common causes of these behaviours [27] but rather as verbal labels given to these psychological systems.

Motivated by the network theory of psychological processes, psychological researchers started to examine network models, statistical models originally developed for network science [28,29] (for example, to investigate networks of websites [30] or networks of interactions between genes and proteins [31]) and explore whether these models could be adapted to estimate and infer psychological networks from the data [20]. In a network, objects are represented by nodes and associations are represented by edges. Among the network models adopted by psychological researchers, the most prominent models are Pairwise Markov Random Fields (PMRFs), such as the Ising Model (for binary variables) and the Gaussian Graphical Model (GGM) (for continuous variables), which describe the conditional associations (edges) between variables (nodes) after controlling for all other variables in the network [17]. In PMRFs, an edge between two variables indicates that these variables are conditionally dependent (i.e., conditionally associated), while the absence of an edge between two variables indicates that these variables are conditionally independent (i.e., not conditionally associated). In summary, the network models used in psychological research provide a system-level perspective of the (conditional) associations established between behaviours, cognitions and emotions (measured by items) and have been used to provide new insights into complex psychological systems such as anxiety and depression [32], post-traumatric stress disorder [33], and substance abuse [34], among others.

### 1.2. Analysis of Psychological Networks

**Figure 1:**
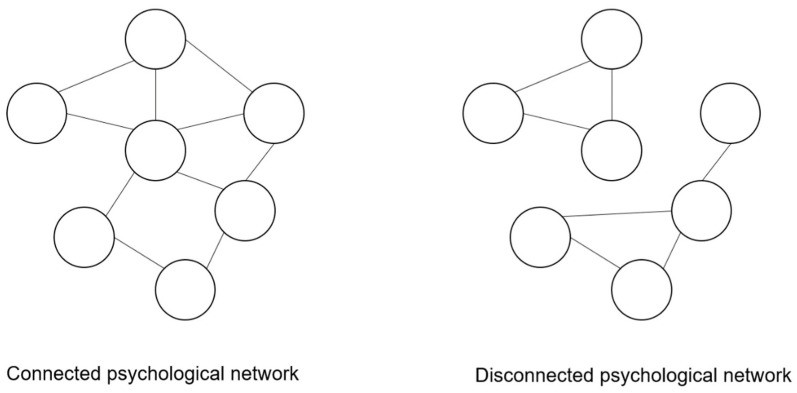
Example of connected and disconnected psychological networks. Note. In the connected psychological network, there is at least one path between every node. In the disconnected psychological network, there is not at least one path between every node, so the disconnected network is composed of smaller isolated networks.

Once a psychological network is estimated from the data, there are four important aspects that need to be evaluated to better understand the complex psychological system. Firstly, to evaluate whether the network is connected or disconnected. On a broader level, a psychological network is considered connected when there is at least one path between every node, while a psychological network is considered to be disconnected when there is not at least one path between every node, so the disconnected network is composed of smaller, isolated networks [35]. An example of connected and disconnected psychological network is displayed in Figure 1.

**Figure 2:**
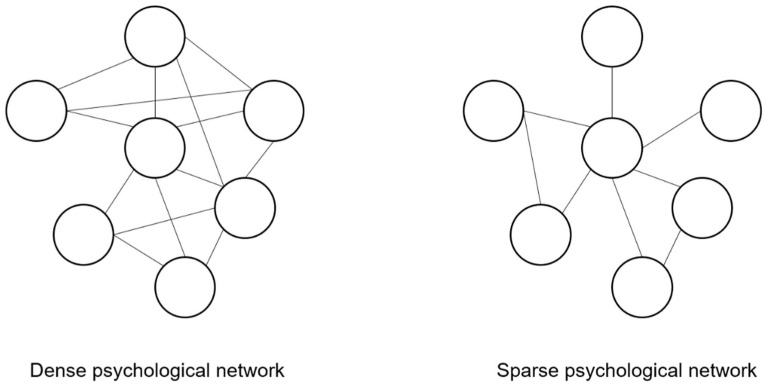
Example of dense and sparse psychological networks. Note. In dense psychological networks, many edges between nodes are present. In sparse psychological networks, only a few edges between nodes are present.

Secondly, to evaluate the network global characteristics. For example, it is important to evaluate whether the network is sparse (only a few edges are present) or dense (many edges are present). This is investigation is important since in dense networks (many edges are present), the importance of individual nodes is less pronounced since all nodes are highly connected, and “intervening” on any node is likely to impact the entire network. Conversely, in the case of sparse networks, the importance of individual nodes is more pronounced since there are a few nodes that are potentially highly connected and they represent possible key targets for interventions [17] (Figure 2).

Thirdly, to evaluate the number of communities in the network. In networks, a community occurs when certain nodes are more strongly connected among themselves compared to other nodes in the network [36]. Algorithms named community detection algorithms were developed to detect communities in networks and certain algorithms have shown good performance in detecting communities in psychological data [37]. One of these algorithms is the walktrap algorithm [38] which employs random walks to find a community configuration that maximises the connections within a set of nodes while minimising the connections of this set of nodes with other nodes from the network.

**Figure 3:**
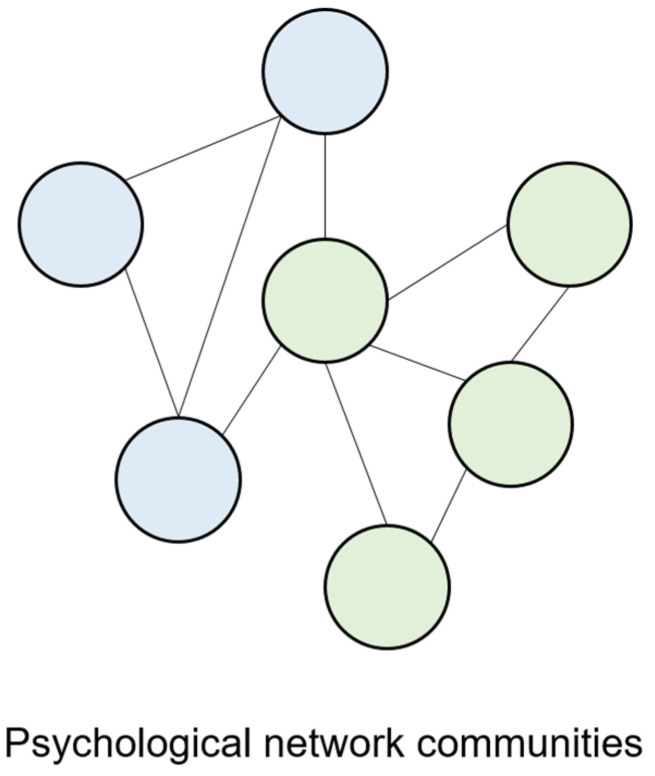
Example of psychological network communities. Note. The green nodes indicate one network community (e.g., depression), while the blue nodes indicate another network community (e.g., social anxiety).

In psychological networks, network communities have been associated with distinct psychological processes (such as distinct psychopathologies). For example, consider a psychological network in which one community represents “depression” (comprised of behaviours, cognitions and emotions associated with depression such as frequent crying, loss of pleasure and sadness) and another community represents “social anxiety” (comprised of behaviours, emotions and cognitions associated with social anxiety such as avoiding going to a party or participating in small groups). In psychological networks, independent communities develop when certain behaviours, cognitions and emotions establish strong mutually reinforcing causal relations among themselves (behaviours associated with depression establish mutually reinforcing causal relations; difficult sleeping → fatigue → sad mood → difficult sleeping) compared to the other behaviours included in the network, clustering together [39]. An example of communities in a psychological network is displayed in Figure 3.

Fourthly, to evaluate the network node centrality. Node centrality indicates the importance of one node in relation to the other nodes in the network. To measure node centrality, centrality measures such as strength centrality have been developed [40]. These measures were traditionally used in network science, for example, in social networks, to indicate which persons (nodes) are more central in the network, having the highest number of friendships (edges) in a social group. In psychological networks, the investigation of node centrality is conducted based on the *centrality hypothesis*. The centrality hypothesis states that the most central nodes are the best targets for intervention since they are believed to represent the most influential behaviours in the psychological network [41]. Moreover, centrality measures have been developed specifically for psychological networks such as bridge centrality [39]. Bridge centrality indicates a node connectivity with more than one community in a psychological network. For example, consider a node that is strongly connected with depression and social anxiety communities, indicating a behaviour that is involved in the occurrence of both depression and social anxiety, constituting a bridge symptom [42] between these two mental disorders. The bridge symptoms were believed to have a fundamental role in the comorbidity observed among several mental disorders (such as major depressive disorder and generalised anxiety disorder) since “the bridge symptoms are connected with one (mental disorder or) another, and comorbidity arises only through connections” between two mental disorders [43] (p. 140).

Despite the development of several centrality measures, the debate regarding the truthfulness of the centrality hypothesis (and the usefulness of centrality measures) in psychological research is ongoing. For instance, centrality measures such as strength centrality (that are calculated based on conditional associations) were shown to be poor substitutes for causal effects (due to reasons such as confounding or collider bias) [44]. Nonetheless, methodological research has indicated that centrality measures such as strength centrality are moderately correlated to the behaviours most suitable for intervention [41] and certain empirical research has also provided support for centrality measures [45].

### 1.3. The Current Study

In Australia, psychological research to understand stress, social support and coping among Aboriginal Australians has been conducted within the latent trait theory framework. To the best of our knowledge, there has been no research that has investigated stress, social support and coping among Aboriginal Australians within the network theory of psychological processes. The network theory of psychological processes can provide new insights into the functioning of this complex psychological system (stress, social support and coping) among Aboriginal Australians by elucidating the conditional associations between behaviours, cognitions and emotions (instead of elucidating associations between the latent traits). This investigation can help inform potential targets for intervention, aiming at reducing stress, increasing social support and increasing coping in Aboriginal populations. To investigate the psychological network of stress, social support and coping among Aboriginal Australians through a network perspective, statistical network models need to be used (instead of models such as factor analytical models that could be used to evaluate stress, coping and social support through a latent variable perspective).

The current study aimsedto investigate the psychological network of stress, coping and social support in an Aboriginal Australian population. Specifically, we aimed to answer four research questions: (1) do stress, coping and social support constitute a connected psychological network in an Aboriginal population? (2) What are the global characteristics of the psychological network of stress, coping and social support in an Aboriginal population? (3) What are the network communities of the psychological network of stress, coping and social support in an Aboriginal population? (4) What is the node centrality of the nodes in the psychological network of stress, coping and social support in an Aboriginal population?

## 2. Materials and Methods

### 2.1. Participants

This study will conduct a secondary analysis of the South Australian Aboriginal Birth Cohort (SAABC) study, a prospective longitudinal birth cohort study which initiated as a randomised controlled trial and was originally designed to assess if an oral-health intervention, which included anticipatory guidance and motivational interviewing, would reduce the prevalence of dental disease among Indigenous children in South Australia. The study was pre-registered and the study protocol can be found in Merrick, et al. [46]. The intervention took place during pregnancy and when children were aged 6, 12 and 18 months for the intervention group, and when children were aged 24 months, 30 months and 36 months for the delayed intervention group.

The study received approval from the University of Adelaide Human Research Ethics Committee (H-057-2010), the Aboriginal Health Council of South Australia (04-09-362), the Government of South Australia and the Human Research Ethics Committees of three participating South Australian hospitals (Flinders Medical Centre: 435-10; Lyell McEwin Hospital: 2010-160; and the Women’s and Children’s Hospital: REC2322/11/13) [46]. Consent from study participants was obtained using the NHMRC Guidelines for Ethical Conduct in Aboriginal and Torres Strait Islander Health Research [46]. Participants were informed that their participation in the study was voluntary and that they could refuse or withdraw at any stage without reason or justification. All participants provided written signed informed consent.

The participants were recruited through referrals from several sources including Indigenous groups, community services and hospitals. Among the original referrals to the study, 41 participants did not enrol due to no longer being interested in participating, responding after the beginning of the study, having a miscarriage, living outside of South Australia, or being unable to be contacted. At baseline, the SAABC included 448 Aboriginal and Non-Aboriginal mothers pregnant with an Aboriginal child at study baseline, comprising two-thirds of the participants eligible for the study (n = 738; based on 2008 estimates of Aboriginal children born from Aboriginal and Non-Aboriginal mothers). One child died in utero and 12 children passed away before the age of two years old. The SAABC sample at baseline was representative of age and socioeconomic position in South Australia [47].

Regarding the study follow-ups, 324 participants were followed when children were aged 2 years, while 324 participants were followed when children were aged 3 years (achieving more than 70% retention rate in both follow-ups). The SAABC is an ongoing longitudinal study and, in addition to the 2, 3, 5 and 7 year-old follow-ups, the families and children are currently participating in the 9 year-old follow-up. For more information on the SAABC, please refer to Jamieson, et al. [47]. The sample used in this secondary analysis includes 367 pregnant Aboriginal women that were part of the SAABC at the study baseline.

The Perceived Stress Scale (aPSS-13) and the Social Support Scale (SSS) were administered as part of a broader questionnaire by research staff (three Indigenous and one non-Indigenous staff) at the study baseline. Considering that it is important that behaviours, cognitions and emotions included in psychological networks are measured with instruments that have their validity and reliability previously established [48], we included instruments that have been previously validated in an Aboriginal Australian population (also using SAABC data) [49]. All procedures performed in studies involving human participants were in accordance with the ethical standards of the institutional and/or national research committee and with the 1964 Helsinki declaration and its later amendments or comparable ethical standards. All participants provided signed informed consent.

### 2.2. Measures

#### 2.2.1. Perceived Stress Scale (aPSS-13)

The Perceived Stress Scale (PSS) evaluates whether a person’s life is perceived as unpredictable, uncontrollable, or overwhelming. Composed of 14 items in its original form (PSS-14) [50], the PSS has two subscales, the Perceived Stress (PS) and Perceived Coping (PC) subscales. The items were responded to on a five-point rating scale (1 = Not at all, 2 = Rarely, 3 = Sometimes, 4 = Fairly often, 5 = Very often). The positively worded items from the PC subscale were not recoded/rescore prior to the analysis. That is, higher scores on the PS items indicate higher perceived stress and higher scores on the PC items indicate higher perceived coping. The PSS-14 was originally included in the SAABC. However, for this secondary analysis, we used a revised version with 13 items (aPSS-13) since a previous validation study (conducted using SAABC data) indicated that this 13-item version (after excluding one misfitting item) was culturally appropriate for an Aboriginal Australian population [49]. The PS (Ω = 0.83; 95% CI [0.80, 0.86]) and PC (Ω = 0.81; 95% CI [0.77, 0.84]) subscales displayed good reliability as indicated by the McDonald’s coefficient Ω [51].

#### 2.2.2. Social Support Scale (SSS)

The Social Support Scale is composed of 4 items that evaluate the emotional, appraisal, instrumental and informational domains of social support [52]. The items were responded to on a five-point rating scale (1 = Not at all, 2 = Rarely, 3 = Sometimes, 4 = Fairly often, 5 = Very often). Since the SSS displayed good psychometric properties for Aboriginal and/or Torres Strait Islanders in a previous validation study (conducted using SAABC data), the instrument was chosen to be included in the network analysis. The SSS (Ω = 0.88; 95% CI [0.84, 0.91]) displayed good reliability.

The aPSS-13 and the SSS items, along with the short labels used throughout this manuscript to refer to each item (e.g., “top” for aPSS Item 9 “…felt you were on top of things?”), are displayed in Supplementary Table S1. The distribution of items scores are displayed in Supplementary Figure S1.

### 2.3. Statistical Analysis

The statistical analysis was conducted with R software version 4.2.0 [53], R packages qgraph version 1.9.2 [54], EGAnet version 1.2.3 [55], powerly version 1.8.6 [56] and NetworkToolbox version 1.4.2 [57], and JASP software version 0.16.4 [58]. The R script used in this study was made available in the Supplementary Materials. Following recommendations on best practices in network psychometrics, pairwise deletion was used when calculating the polychoric correlation coefficient matrix used as input for the network models [59].

#### 2.3.1. Network Model and Estimation

The network model used was the Gaussian Graphical Model (GGM) [60]. The GGM models the precision matrix (i.e., inverse of the variance covariance matrix) such that (after standardizing the precision matrix and reversing the sign) nodes represent items and edges represent partial correlation coefficients [61] between items. Thus, an edge indicates conditional dependence (after conditioning on the entire set of variables) between two items, while the absence of an edge indicates conditional independence [60]. Considering that the SDQ items are ordinal polytomous items, the network was estimated based on polychoric correlation coefficients [62]. Since partial correlations will rarely be exact zeros, to estimate a sparser network that can be more easily interpreted, GGM was estimated using a penalised maximum likelihood estimation, namely the least absolute shrinkage and selection operator (LASSO) [63]. The LASSO turning parameter selection was based on minimising the Extended Bayesian Information Criteria (EBIC) [64,65]. Missing data for individual items ranged from 0.0% to 1.1%, so multiple imputation was not required [66]. The final network was plotted with the Fruchterman–Reingold algorithm [67], which arranges the nodes according to the strength of their connections (edge weights) so nodes with stronger connections are displayed more closely.

#### 2.3.2. Sample Size Estimation

To calculate the sample size needed for estimating the network model, we used a Monte Carlo simulation method [56]. We investigated the sample size needed to estimate a GGM with 17 nodes (the same number of nodes of the network evaluated in this study) and edge density of 0.4 (compatible with other psychological networks [68]) reaching a sensitivity (the number of true estimated edges (true positives) over the total number of estimated edges (true positives + false negatives)) of 60% across 80% of all cases (power). Sample size requirements based on power analysis for model estimation in observational data collected from Indigenous populations, however, need to be interpreted cautiously due to two considerations. Firstly, Hernán [69] argued that the practice of power analysis with observational data is misguided since the goal of the analysis is not to “detect” a parameter (a network edge), considering that the parameters “are not binary signals that are either detected or undetected” but rather “are numerical quantities that need to be estimated”. Hence, instead of focusing on the power of observational studies to “detect” a specific parameter of interest, the solution is to estimate the model parameters as precisely as possible from multiple studies, so that estimates can later be pooled through meta-analytical techniques. Meta-analytical techniques are already available for network models [70]. Secondly, despite the importance of understanding sample sizes that would achieve optimal estimation precision, studies involving Indigenous populations frequently have smaller sample sizes, since Indigenous groups usually comprise a small proportion of the overall population and due to the difficulties in recruiting Indigenous participants [71,72].

#### 2.3.3. Network Global Characteristics

To investigate network global characteristics, we examined the (1) edge density. The edge density is the number of edges present in the network divided by the number of all potential edges in the network [73]. We also examined the (2) global clustering coefficient. The clustering coefficient evaluates whether adjacent nodes connected to a node (i.e., the “neighbours” of a node) will be connected among themselves [74]. The global clustering coefficient, which was generalised to weighted networks, measures node clustering across the entire network [75]. Networks with higher clustering coefficients also have a higher density [74].

#### 2.3.4. Network Communities

The walktrap algorithm was used to identify the communities in the network [38]. To evaluate the uncertainty regarding the identified communities, the walktrap algorithm was applied to 2500 parametric bootstrap samples to calculate a 95% CI for the number of communities [76]. Finally, to measure community connectivity, we calculated the Root Mean Squared Edge Weight (RMSEW) between communities and compared them to values within communities.

#### 2.3.5. Network Local Characteristics

To evaluate network local characteristics, we investigated centrality measures [77]. Firstly, we evaluated node strength [74], which is the sum of the absolute values of the edge weights established by a node with all other nodes in the network (i.e., how many connections a node has and how strong these connections are) [78]. Secondly, we evaluated eigencentrality, which measures whether a node is connected to other nodes with many connections of their own. The eigencentrality evaluates the quality of the connections [79] since a node connected to other nodes with many connections (“popular” nodes) is more important than one connected to nodes without many connections (“unpopular” nodes). Furthermore, eigencentrality was shown to the best predictor of nodes with the highest Average Causal Effect (ACE) on other nodes compared to other centrality measures [44]. Thirdly, we evaluated bridge strength, which is the sum of the absolute values of the edge weights established by a node with other communities besides the community that the node originally belongs to (i.e., how many connections a node has and how strong these connections are with communities that the node does not belong to). We did not evaluate betweenness [80] or closeness centrality [80] since these metrics were originally developed to evaluate distances between nodes (e.g., the number of airports on a route between two cities). However, psychological networks are constituted of conditional associations and interpreting conditional associations as distances is not conceptually meaningful, hindering the original interpretation and precluding the use of betweenness and closeness centrality measures in psychological networks [17,81]. Finally, we inspected the Minimum Spanning Tree (MST) [82]. The MST is a reduced network that connects all nodes (without cycles) using the edges that carry the most information. Thus, the MST identifies the “backbone structure” of the network and complements centrality measures by focusing only on the most essential edges [77]. The MST was calculated based on the Gower [83] distance of partial correlations [77] through the Kruskal [84] algorithm.

#### 2.3.6. Network Precision and Stability

To evaluate the sample edge weights’ precision, we employed 2500 non-parametric bootstrap samples to derive point estimates and data-mined edge weights [85]. Furthermore, we used the correlation stability coefficient (CS coefficient) to measure the stability of edge weights and strength centrality across subsamples. To ensure that estimated differences in edge weights and strength centrality represent true differences in the network (rather than sampling variation), a simulation study showed that CS coefficient values should not be below 0.25 and preferably higher than 0.50 [86].

## 3. Results

The sample was composed of Aboriginal pregnant women with an average age of 24.9 years (SD = 5.9, range = 17–43), predominantly educated up to high school (73%), not employed (87%) and living in areas pertaining to the lowest quintile of the Index of Relative Socio-Economic Advantage and Disadvantage (IRSAD) (54%). These demographic characteristics indicated a population that was largely socio-economically disadvantaged. All demographic characteristics are displayed in Table 1.

### 3.1. Network Model and Estimation

**Figure 4:**
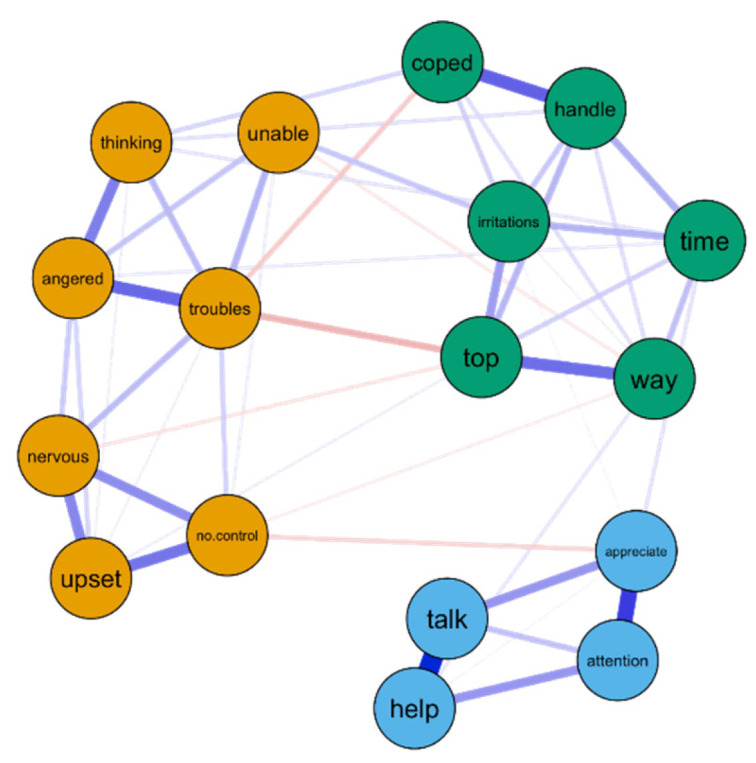
Network of stress, coping and social support in an Aboriginal population. Note. Positive edges are displayed as blue lines and negative edges are displayed as red lines. Edge weights are represented by the thickness and saturation of the edges.

The sample size needed to estimate a GGM with 17 nodes and an edge density of 0.4 reaching a sensitivity of 60% across 80% of all cases was 2,248 participants (the limitations regarding not achieving the optimal sample size for model estimation are discussed in the Discussion section (Section 4)). The structure of the network is displayed in Figure 4.

### 3.2. Network Characteristics

**Figure 5:**
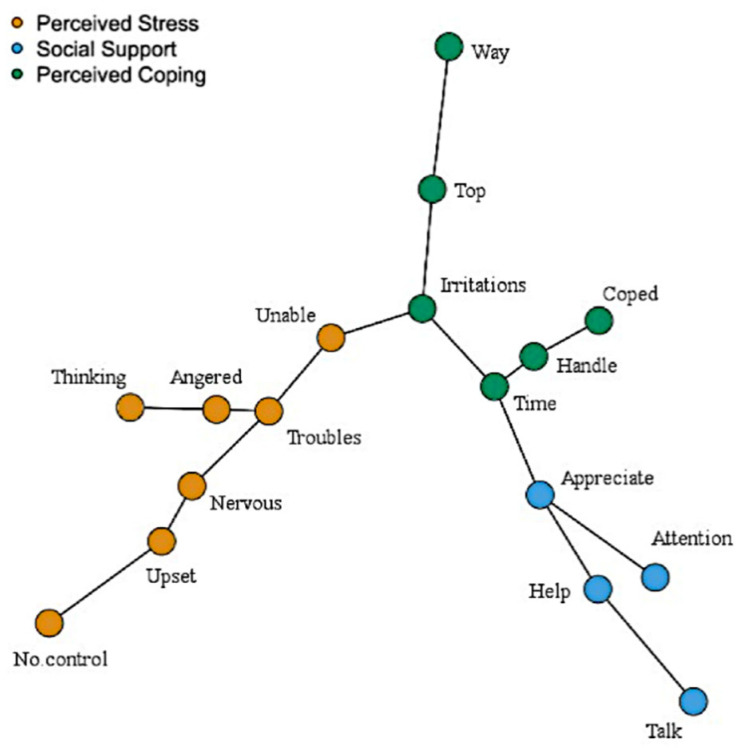
Minimum Spanning Tree (MST) of stress, coping and social support in an Aboriginal population.

Regarding the global characteristics, the network had an edge density of 0.53 and a global clustering coefficient of 0.61. The MST is displayed in Figure 5.

### 3.3. Network Communities

The walktrap algorithm indicated that the network was composed of three distinct communities of stress, coping and social support comprising the exact same items of the corresponding subscales (Perceived Stress subscale and Perceived Coping subscale (from the aPSS-13), and the Social Support Scale, respectively). The bootstrap samples had a median number of three communities (95% CI [2.993, 3.006]). The (root mean squared) edge weight was higher between the stress and coping communities (M = 0.08, SD = 0.09), followed by the coping and social support communities (M = 0.04, SD = 0.06) and the stress and social support communities (M = 0.04, SD = 0.06). As expected, these values between communities were smaller than values found within the communities of stress (M = 0.18, SD = 0.20), coping (M = 0.18, SD = 0.19) and support (M = 0.34, SD = 0.36).

### 3.4. Node Centrality

**Figure 6:**
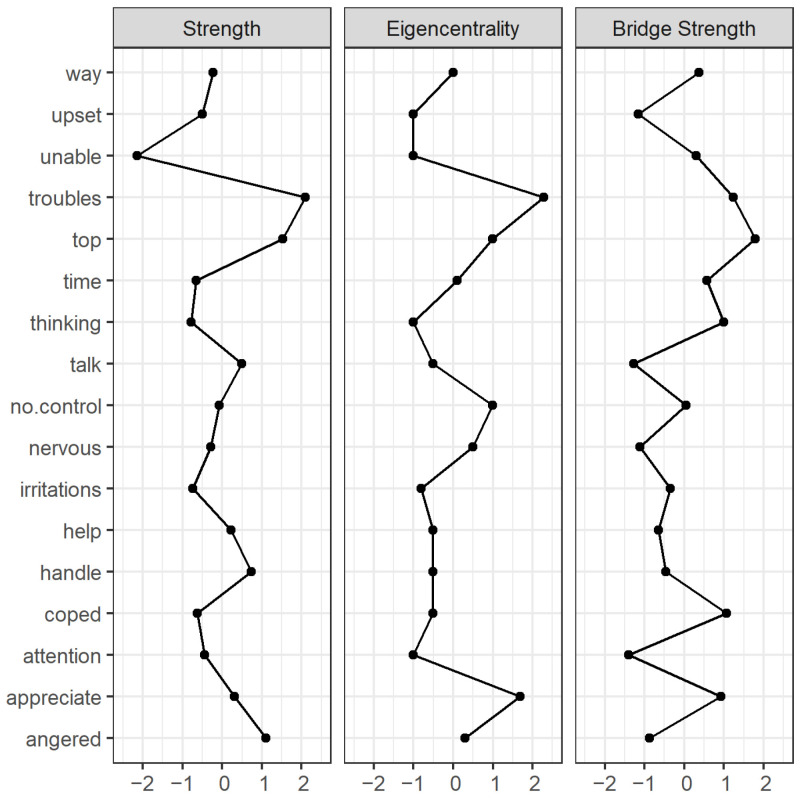
Centrality plot of stress, coping and social support in an Aboriginal population.

The node with the lowest strength centrality and eigencentrality was aPSS Item 7 ‘unable’ (“…felt unable to cope with all the things that you had to do?”) (Sc = −2.143; Ec = −2.008), while the node with the highest strength centrality and eigencentrality was aPSS Item 13 ‘troubles’ (“…felt troubles were piling up so high that you could not deal with them?”) (Sc = 2.090; Ec = 2.224). The node with the highest bridge strength was aPSS Item 13 ‘top’ (“…felt you were on top of things?”) (Bc = 1.795), followed by the aPSS Item 13 ‘troubles’ (Bc = 1.233). The centrality indices are displayed in Figure 6.

All centrality indices are displayed in Table 2.

### 3.5. Network Precision and Stability

The point estimates of sample edge-weights were consistent with the mean edge weights estimated from 2500 bootstrap samples. The data-mined edge weights are displayed in Supplementary Figure S2. The CS coefficients for edge-weights (CSe = 0.67) and node strength (CSs = 0.52) were above the minimum threshold of 0.25 and the ideal threshold of 0.50. That is, 67% is the maximum proportion of the sample that can be randomly removed so there is a 95% probability that the correlation of the edge-weights between the original sample and the subsamples is 0.7 or higher. Similarly, 52% is the maximum proportion of the sample that can be randomly removed so there is a 95% probability that the correlation of the node strengths between the sample and subsamples is 0.7 or higher.

## 4. Discussion

The aim of the present study was to investigate the psychological network of stress, coping and social support in Aboriginal Australians. The current study showed that: (1) stress, coping and social support constituted a connected psychological network in an Aboriginal population; (2) the global network characteristics indicated that the psychological network of stress, coping and social support was as dense as other psychological networks; (3) three communities of stress, coping and social support were identified; (4) at the centre of the network were the troubles experienced by the Aboriginal pregnant women (“felt troubles were piling up so high that you could not deal with them?”), constituting a bridge between perceived stress and coping processes in this population. The implications are discussed in the next paragraphs.

### 4.1. Network Global Characteristics

The presence of at least one path between every node (every behaviour, cognition and emotion) of the network indicates that stress, coping and social support form a connected psychological network [35]. The communities of stress, coping and support could have been completely isolated from one another. However, in our study, a possible path existed between every pair of nodes, reinforcing that stress, coping and social support behaviours, cognitions and emotions do interact as a single psychological network in an Aboriginal Australian population.

The network edge density of 0.53 was comparable to values found in other psychological networks, such as network models of post-traumatric stress disorder (0.67) [87] and mild cognitive impairment (0.78) [68]. The global clustering coefficient of 0.61 was also similar to values found in other psychological networks, such as the global clustering coefficient of 0.49 and 0.56 reported in two networks of schizotypal symptoms [88]. That is, the density of the stress, coping and social support network is similar to the density of other psychological networks reported in the literature.

### 4.2. Network Communities

The visual inspection of the network indicated three distinguishable communities of nodes, comprised of items measuring stress (from the aPSS-13 Perceived Stress subscale), coping (from the aPSS-13 Perceived Coping subscale), and social support (from the SSS). These three communities were confirmed by the walktrap algorithm and replicated in all bootstrapped samples.

The examination of edge weights indicated that primarily negative connections were found between the stress and coping communities. These associations are consistent with Lazarus’s [89] seminal theory that the perception of sufficient coping resources can change the appraisal of a situation as threatening and, consequently, diminish the stress response. That is, when respondents perceived that they had coping resources to deal with their life difficulties, they also perceived themselves as less stressed. Since Lazarus [89] published his work, an extensive body of research provided further evidence that coping strategies can diminish the psychological and physiological effects of stress [90]. In our study, the connection between stress and coping communities was the strongest in the network.

In addition, positive connections were found between the social support and coping communities and negative connections between social support and stress communities. These associations were also consistent with theory. Seminal research by Cohen and Wills [91] showed that social support (a) increases coping, by providing external resources to deal with a given problem and (b) reduces stress, since the support provided decreases the perception of how stressful the situation is. The protective (and buffering) effects of social support on stress were later confirmed by a large body of empirical research [92].

Node centrality: The most central node of the network was aPSS Item 13 ’troubles’ (“…felt troubles were piling up so high that you could not deal with them?”) as indicated by the strength and eigencentrality. This node was negatively conditionally associated with the second most central node of the network, aPSS Item 9 ’top’ (“…felt you were on top of things?) and, unsurprisingly, had also a strong negative conditional association with aPSS Item 4 ’coped’ (“…coped well with important changes in your life?”). Furthermore, the aPSS Item 9 ’top’ and Item 13 ’troubles’ had also the two highest bridge strength, respectively, indicating that these behaviours constituted the main bridge between the psychological processes of perceived stress and perceived coping in an Aboriginal population. Hence, these findings suggest that being overwhelmed by problems (troubles piling up high) is central to the Aboriginal participant’s perceptions of stress and directly influences their perception of being able to cope with life.

The inspection of the MST reinforced that “troubles piling up so high”, being “unable to cope with all the things” and being “(un)able to control irritations” were central to the psychological network. The branches of the MST often include nodes that “can be interpreted as having some commonalities in meaning” [77] (p. 8). For example, in our study, one branch contained the nodes of feeling “on top of things” and “things were going your way”. The similarity comes from the fact that if an individual is feeling on top of things, she/he most likely feels that things are going their way. Another example is the branch with the nodes “coped well with important changes” and “felt able to handle your personal problems”. Once again, coping with important changes in life in many cases logically imply being able to handle personal problems. A psychological network should include autonomous causal components [17]. Hence, the inspection of MST branches can potentially also be useful to indicate items that are too similar in content to be included in the network, resembling the classical psychometric concept of local dependence [93].

The centrality of being overwhelmed by problems possibly reflects the context of Aboriginal people in contemporary Australia. As a consequence of colonisation and subsequent decades of marginalisation, Aboriginal Australians are profoundly disadvantaged in several key areas, including employment, income, educational attainment, and health [94]. For example, the risk of being exposed to stressful life events is two to five times greater for Aboriginal Australians compared to non-Aboriginal Australians [95]. Thus, it is possible that the disproportionate number of stressful life events experienced by Aboriginal people is what makes being overwhelmed by problems, which is central to their experience of stress, coping and support.

The conditional associations between the four different classes of supportive behaviour (instrumental, informational, emotional, and appraisal) were also consistent with previous social support literature. Firstly, we found stronger conditional associations between informational and instrumental support and between emotional and appraisal support. Shakespeare-Finch and Obst [96] explained that these conditional associations occur because informational support is, in fact, a form of instrumental support. That is, providing useful information (informational support) can often directly help someone solve a task (instrumental support). Similarly, appraisal support can be seen as a form of emotional support, since providing encouraging feedback or expressing high regard is often perceived as an emotionally supportive act. Secondly, we found that all forms of social support were conditionally associated, constituting a single community of behaviours, cognitions and emotions (instead of multiple communities). This finding is consistent with previous literature. For instance, in a seminal work, House [97] proposed social support as a unique (i.e., “unidimensional”) domain that encompasses the four classes of supportive behaviour, a position which later became consensus in the social support literature [98].

### 4.3. Network Precision and Stability

The network precision and stability across bootstrap samples were adequate. Firstly, sample edge weights were consistent with point estimates of the bootstrap samples. Secondly, edge weights from subsamples retained a 0.7 correlation with the original weights even after more than 60% of the sample was excluded. That is, it is likely that observed differences in estimated edge weights represent true differences in edge weights rather than merely sampling error. The strength centrality indices also displayed adequate stability [86].

### 4.4. Theoretical Contributions

One main theoretical contribution is that the study findings indicated that behaviours, cognitions and emotions related to stress, coping and social support do constitute a connected psychological network in Aboriginal Australians. The theoretical advantage (over previous studies) is that the network theory of psychological processes provides a conceptualisation of what is “stress”, “coping” and “social support” in an Aboriginal population. According to the network theory of psychological processes, stress, coping and social support refer to three distinct communities of mutually causally reinforcing behaviours, cognitions and emotions, and these communities were empirically identified in the data. Hence, it is possible to conceptualise stress, coping and social support in an Aboriginal population without the need to rely on latent traits (unobservable psychological process) that would be the common cause behind these behaviours, cognitions and emotions. This is advantageous since, despite the usefulness of latent variables as a statistical procedure, the existence of these latent traits in the real world (their ontological stance) is not always clear [99]. For example, according to the latent trait theory, social support would be the cause of instrumental support (the act of helping solve a task) or informational support (the act of providing information to help solve a task). However, is not clear what social support *is.* In our understanding (and according to the network theory of psychological processes), “social support” is best conceptualised as a verbal label given to the mutually reinforcing associations established between the four different classes of supportive behaviour (informational, instrumental, emotional, and appraisal). That is, individuals who experience instrumental support (e.g., direct help from friends to solve problems) will also receive more informational support (e.g., friends will also give information on how to solve problems) and will experience more appraisal support (e.g., will feel more appreciated since friends are helping them) and so forth. Thus, the network theory of psychological processes and the empirical investigation through network models provide a theoretical conceptualisation of the process of stress, coping and social support in an Aboriginal population.

The processes of stress, coping and social support have also been investigated in Indigenous populations from other countries. The intergenerational trauma due to colonisation and persistent socioeconomic disadvantages have impacted Indigenous peoples all over the world, leading to poorer psychological outcomes in terms of depression, anxiety, posttraumatic stress disorder, and substance abuse, among others [5]. Nonetheless, despite being impacted by historical trauma and land dispossession, “a remarkably large proportion [of Indigenous people in Canada] show considerable resilience” and have relied upon cultural strengths and practices that promote “intergenerational resilience” [5]. As another example, instead of a Western-centric stress-coping model (which focuses on individual coping behaviours), American Indians’ and Alaskan Natives’ coping strategies include connection to community, spirituality, traditional healing practices and enculturation, and are better understood through an “Indigenist stress-coping model” [100]. These successful culture-based ways of coping are employed by Indigenous peoples all across the world, such as Bedouin peoples in Israel, who have relied upon (and “rediscovered”) traditional healthy foods to cope with chronic illness [101]. Social support has also been considered one of the main psychosocial determinants of Indigenous people’s ability to thrive despite largely unfavourable circumstances [102,103].

However, previous studies have investigated the processes of stress, coping and social support in Indigenous populations worldwide qualitatively [101] or quantitatively with latent variables models [102]. Another contribution of our study is that the use of network psychometric models can inform the conditional associations established between behaviours, cognitions and emotions (item scores), instead of the associations established between latent traits (total scores, subscale scores and factor scores). Hence, it is possible to generate hypotheses regarding behaviours, emotions and cognitions which are central to the network and can potentially be intervened upon. For instance, our study showed the negative conditional association between “problems piling up high” and “feeling on top of things” as being central to the Aboriginal experience, and that these two behaviours were the main bridge between perceived stress and perceived coping. In case only total scores (instead of item scores) were examined, these conditional associations between behaviours, emotions, and cognitions would have been concealed.

### 4.5. Limitations

The network theory of psychological process postulates that psychological networks comprise mutually causally reinforcing behaviours, cognitions and emotions. However, the interpretation of network models should be conducted with caution, since these models can only inform conditional associations (rather than causal effects). The reason is that estimated conditional associations between nodes can be spurious in the presence of unmeasured confounders [104] or biased when conditioning on a collider [105]. For an in-depth discussion of how networks relate to causal models, such as models represented by direct acyclic graphs (DAGs), see Ryan, Bringmann [106]. We estimated psychological networks based on cross-sectional data, imposing further constraints on any interpretation regarding causality (due to “reverse-causation”) [107]. Furthermore, psychological networks estimated from cross-sectional data can only inform differences between individuals, and this does not necessarily are equivalent to processes at an individual level (e.g., trouble pilling up would cause an individual to not feel on top of things) [17]. Despite these limitations, psychological networks estimated from cross-sectional data have their usefulness in generating hypotheses about the data-generating structure and providing a clear theoretical framework to explain how behaviours, cognitions and emotions are conditionally associated and how they cluster together into distinct communities. These hypotheses are “a major asset in a field like psychology, where strong causal theory is sparse and the identification of DAGs often appears a bridge too far” [108] (p. 25). Future research should investigate and confirm the hypothesis generated in this study (e.g., that the perception of troubles piling up has a causal impact on the perception of feeling on top of things) using causal inference models in other Aboriginal Australian samples.

Another limitation was that, while the current study included a large sample considering Indigenous research [71,72], a larger sample (n > 2000) would be required for optimal estimation of the network model parameters. Hence, it is possible that not all true network edges were captured during model estimation. However, the fact that the study sample size was not ideal should not, by any means, discourage model estimation and interpretation of findings. Hernán [69] discusses how power analysis with observational data can be misguided since the aim of the study is not “to detect” or “not to detect” a parameter but rather to estimate the parameter as precisely as possible. The estimated parameter is of scientific interest and can be pooled in the future with parameters from other studies through meta-analytical methods, generating more precise evidence to inform interventions. Future studies should estimate psychological networks of stress, coping and social support in other Aboriginal Australian samples and meta-analytical methods can estimate a pooled meta-analytic network structure of stress, coping and social support in Aboriginal populations [70].

Other limitations include that the sample was composed of Aboriginal pregnant women, so the generalisability of these results to other Aboriginal groups (including Aboriginal men) is unclear. Future studies should evaluate psychological networks in samples that include Aboriginal men and women. Furthermore, although the number of participants referred to the study that did not enrol was small (41 out of the 448 participants enrolled at the study baseline), it is possible that the participants that did not enrol had distinct characteristics from those who enrolled. In case the failure to enrol did not occur completely at random (i.e., “missing completely at random”) [109], it is possible that the estimated network parameters are different from the psychological network in the population (but not necessarily result in them being different [110]). Future studies should try to replicate these results in other samples from the same Aboriginal population.

## 5. Conclusions

Over the last decades, researchers have aimed to understand the influence of psychosocial factors on the health inequalities experienced by Aboriginal Australians. In this study, we showed that three communities (stress, coping and social support) of individual behaviours, emotions and cognitions were identified in pregnant Aboriginal women. Furthermore, these communities constituted a connected psychological network, implicating that interventions on one behaviour (and, preferably, targeted to central behaviours) can potentially spread and influence the whole network. The network structure provided a hypothesis of how communities of behaviours, cognitions and emotions related to stress, coping and social support are conditionally associated in a population of pregnant Aboriginal women, paving the ground for future research with causal models.

## Figures and Tables

**Table 1:** Sociodemographic characteristics.

Sociodemographic Characteristics	n	%
**Age**		
17/22	125	34.0%
23/27	109	29.7%
28/43	115	31.4%
Missing	18	4.9%
**Sex**		
Female	367	100.0%
Male	0	0.0%
Missing	0	0.0%
**Education**		
High school or less	266	73.3%
TAFE or university	98	26.7%
Missing	0	0.0%
**Employment**		
Yes	45	12.4%
No	316	87.3%
Missing	1	0.3%
**IRSAD**		
1st	6	1.6%
2nd	16	4.4%
3rd	82	22.3%
4th	64	17.4%
5th	198	54.0%
Missing	1	0.3%

Note. Numbers and percentages. TAFE, Technical and Further Education (trade school/college).

**Table 2:** Centrality measures per variable.

Variable	Network		
	Strength	Eigencentrality	Bridge Strength
Angered	1.098	1.318	−0.880
Appreciate	0.312	0.026	0.925
Attention	−0.436	−0.469	−1.405
Coped	−0.634	−0.606	1.073
Handle	0.739	0.328	−0.471
Help	0.225	0.246	−0.650
Irritations	−0.747	−1.046	−0.356
Nervous	−0.283	−0.002	−1.109
No. control	−0.078	−0.221	0.046
Talk	0.494	0.548	−1.274
Thinking	−0.782	−0.386	0.990
Time	−0.659	−0.771	0.568
Top	1.522	1.455	1.795
Troubles	2.090	2.224	1.233
Unable	−2.143	−2.008	0.296
Upset	−0.493	−0.414	−1.158
Way	−0.225	−0.221	0.376

Note: Centrality indices were calculated as standardised z-scores.

## Data Availability

Data cannot be shared publicly because of its sensitive nature. The study participants constituted a significant proportion of the Aboriginal and Torres Strait Islander community in South Australia and the release of data could lead to the participants’ identification. Data are available from the Aboriginal Research Advisory Committee of the Indigenous Oral Health Unit (Email: iohu@adelaide.edu.au. Tel.: +61-8-8313-4611) for researchers who meet the criteria for access to confidential data.

## Supplementary Materials

The following supporting information can be downloaded at: https://www.mdpi.com/article/10.3390/ijerph192215104/s1, Table S1: Item content and labels; Figure S1: Item score distribution; Figure S2: Bootstrapped data-mined edge weights of the psychological network of stress, coping and social support in an Aboriginal population.
