# Supplementary material for: Psychological Network of Stress, Coping and Social Support in an Aboriginal Population
Source: Int J Environ Res Public Health. 2022 Nov 16;19(22):15104. doi: 10.3390/ijerph192215104 (PMC9690794; doi:10.3390/ijerph192215104)
Supplement: Supplementary file 1 [file ijerph-19-15104-s001.zip › ijerph-1983216-supplementary.pdf]

Supplementary Table S1. Item content and labels.

Item Number	Content	Instrument/Subscale	Label
1	...felt upset because of something that happened?	aPSS13/ Perceived Stress	upset
2	...felt like you couldn't control the important things in your life?	aPSS13/ Perceived Stress	no.control
3	...felt nervous or stressed?	aPSS13/ Perceived Stress	nervous
4	...coped well with important changes in your life?	aPSS13/ Perceived Coping	coped
5	...felt able to handle your personal problems?	aPSS13/ Perceived Coping	handle
6	...felt things were going your way?	aPSS13/ Perceived Coping	way
7	...felt unable to cope with all the things that you had to do?	aPSS13/ Perceived Stress	unable
8	...felt able to control irritations in your life?	aPSS13/ Perceived Coping	irritations
9	...felt you were on top of things?	aPSS13/ Perceived Coping	top
10	...felt angered because of things that happened outside of your control?	aPSS13/ Perceived Stress	angered
11	...found yourself thinking about all the things that you have to do?	aPSS13/ Perceived Stress	thinking
12	...felt able to control how you spend your time?	aPSS13/ Perceived Coping	time
13	...felt troubles were piling up so high that you could not deal with them?	aPSS13/ Perceived Stress	troubles
1	There are people in my life who pay attention to my feelings and problems	Social Support Scale	attention
2	There are people in my life who appreciate what I do	Social Support Scale	appreciate
3	There are people in my life who I can get help from if I need it	Social Support Scale	help
4	There are people in my life who I can talk to about how to handle things	Social Support Scale	talk

Note. All aPSS-13 items started with the sentence "How often during the LAST YEAR have you...".

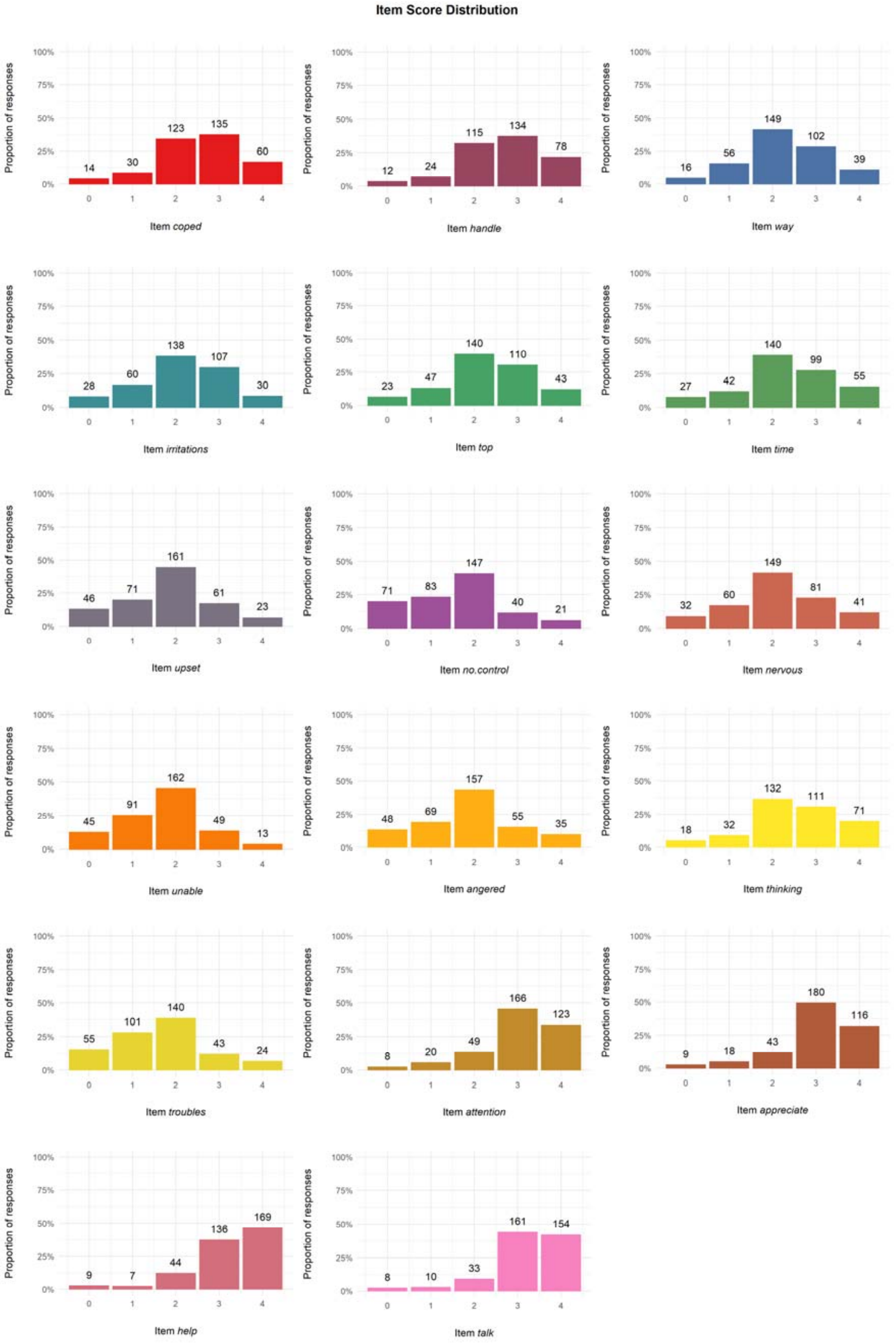

Supplementary Figure S1. Item score distribution. Note. The x-axis indicates the item response categories ranging from 0 to 4. The y-axis indicates the proportion of individuals that endorsed each of the response categories.

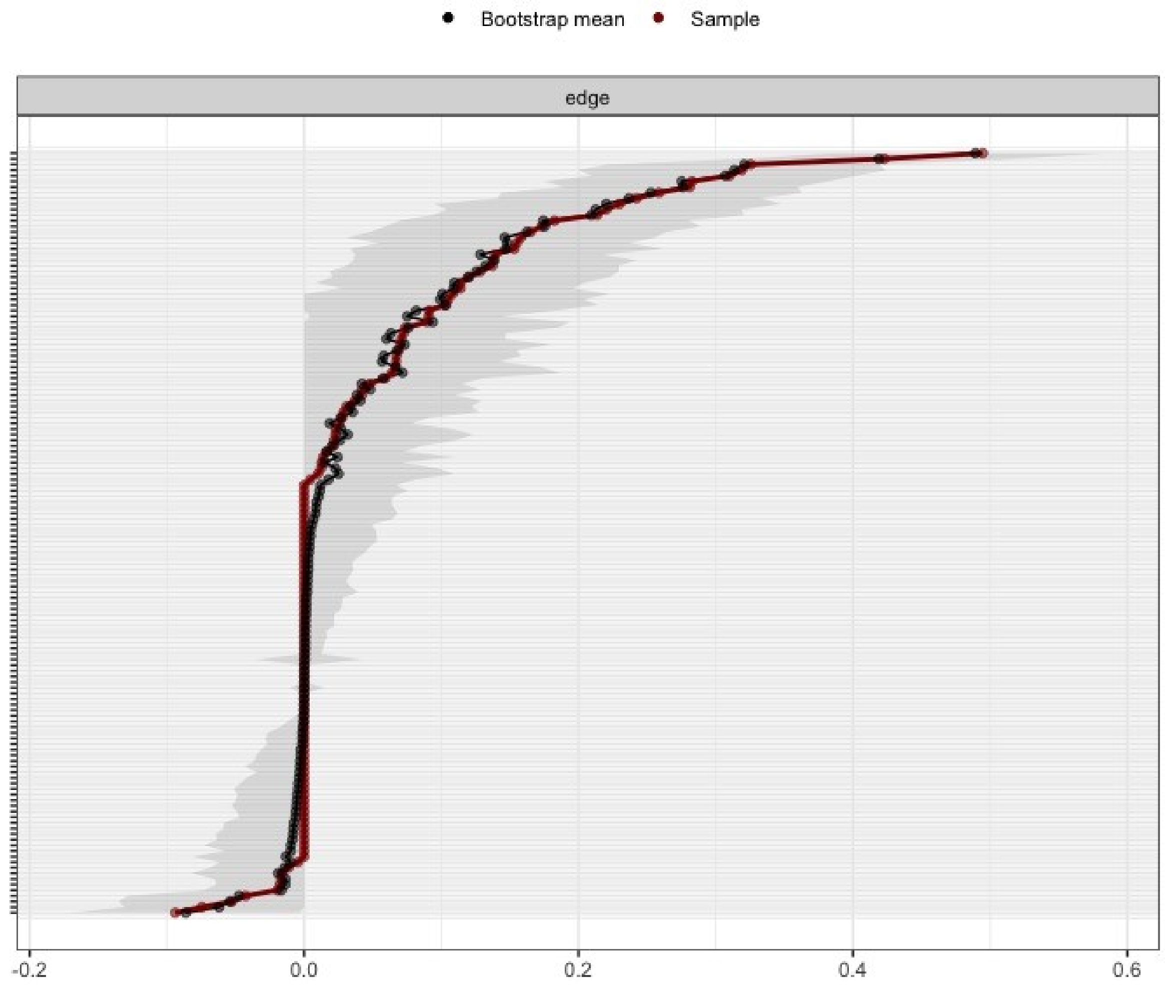

Supplementary Figure S2. Bootstrapped data-mined edge weights of the psychological network of stress, coping and social support in an Aboriginal population. *Note*. The x-axis indicates the edge-weight and the y-axis indicates the edges. The red line represents the edge weights estimated from the sample, while the black line represents the mean edge weights estimated from 2500 bootstrap samples. The grey area displays the data-mined edge weights.
